# Prediction of exercise sudden death in rabbit exhaustive swimming using deep neural network

**DOI:** 10.1186/s12938-021-00925-0

**Published:** 2021-08-30

**Authors:** Yao Zhang, Yineng Zheng, Menglu Wang, Xingming Guo

**Affiliations:** 1 Key Laboratory of Biorheology Science and Technology, Ministry of Education, College of Bioengineering, Chongqing University, Chongqing, 400044 China; 2 Department of Radiology, The First Affiliated Hospital of Chongqing Medical University, Chongqing, 400016 China

**Keywords:** Heart sounds, Deep learning, Exhaustive swimming experiment, Exercise sudden death

## Abstract

**Background and objective:**

Moderate exercise contributes to good health. However, excessive exercise may lead to cardiac fatigue, myocardial damage and even exercise sudden death. Monitoring the heart health has important implication to prevent exercise sudden death. Diagnosis methods such as electrocardiogram, echocardiogram, blood pressure and histological analysis have shown that arrhythmia and left ventricular fibrosis are early warning symptoms of exercise sudden death. Heart sounds (HS) can reflect the changes of cardiac valve, cardiac blood flow and myocardial function. Deep learning has drawn wide attention because of its ability to recognize disease. Therefore, a deep learning method combined with HS was proposed to predict exercise sudden death in New Zealand rabbits. The objective is to develop a method to predict exercise sudden death in New Zealand rabbits.

**Methods:**

This paper proposed a method to predict exercise sudden death in New Zealand rabbits based on convolutional neural network (CNN) and gated recurrent unit (GRU). The weight-bearing exhaustive swimming experiment was conducted to obtain the HS of exercise sudden death and surviving New Zealand rabbits (*n* = 11/10) at four different time points. Then, the improved Viola integral method and double threshold method were employed to segment HS signals. The segmented HS frames at different time points were taken as the input of a combined CNN and GRU called CNN–GRU network to complete the prediction of exercise sudden death.

**Results:**

In order to evaluate the performance of proposed network, CNN and GRU were used for comparison. When the fourth time point segmented HS frames were taken as input, the result shows that the proposed network has better performance with an accuracy of 89.57%, a sensitivity of 89.38% and a specificity of 92.20%. In addition, the segmented HS frames at different time points were input into CNN–GRU network, and the result shows that with the progress of the experiment, the prediction accuracy of exercise sudden death in New Zealand rabbits increased from 50.98 to 89.57%.

**Conclusion:**

The proposed network shows good performance in classifying HS, which proves the feasibility of deep learning in exploring exercise sudden death. Further, it may have important implications in helping humans explore exercise sudden death.

## Background

Exercise sudden death has attracted widespread attentions due to the difficult prediction, short onset time and high mortality [1, 2]. Cardiac function reflects the ability of the heart to work, and some indicators related to cardiac function such as ejection fraction and systolic blood pressure have been confirmed to alert to sudden death caused by excessive exercise [3, 4]. Therefore, it is of great significance to pay attention to the changes of heart function during exercise for guiding people to exercise scientifically and preventing exercise sudden death.

Exercise sudden death refers to non-traumatic accidental death occurring during exercise or within 24 h after exercise [3]. The temporary decrease in cardiac function caused by high-intensity, long-term and multi-round exercise is called cardiac fatigue and usually precedes exercise sudden death [5, 6], where multi-round exercise refers to repeated exercise. If the state of reduced cardiac function is not restored within 24 to 48 h, it will lead to a series of abnormalities, such as systolic and diastolic dysfunction, myocardial contractility reduction, cardiac burden increment, and even exercise sudden death [7].

The pathogenesis of sudden death caused by excessive exercise is still unclear [8]. Unexplained exercise sudden death is of great interest during exercise and competition [3, 9–11]. Recent theoretical developments have shown that exercise intensity and duration are important factors [12, 13]. Animal experiments have been widely used to study the changes of cardiac function during exercise. The experimental subjects mainly include rats, rabbits, sheep, canine, swine and horses, and the experimental methods are mostly running or swimming [8, 13–18]. Rabbits are often used to explore various cardiovascular diseases because their myocardial is similar to human’s in function [18, 19] and swimming can significantly affect the cardiac function with less emotional involvement [20]. Therefore, it is a way to study the changes of cardiac function during exercise to establish rabbit model through the exhaustive swimming experiment.

Clinical methods have been widely used to monitor cardiac status during exercise. Some researchers have found that indexes related to systolic function decline after prolonged exercise by echocardiography, electrocardiogram and/or biochemical indicators [13, 16]. Moreover, the previous studies have shown that intense exercise may lead to pathological heart remodeling and ultimately to myocardial fibrosis, which affects the diastolic and systolic function of the heart [7]. As a safe and non-invasive diagnostic method, heart sounds (HS) can reflect the diastolic and systolic functions of the heart from the perspective of myocardial inotropism [21]. To date, the research on exercise sudden death using HS has been rarely reported yet. Therefore, it is necessary to analyze the HS of rabbits during exercise to find the changes of cardiac function before exercise sudden death.

Machine learning is an effective tool for heart sound classification and prediction. Some studies on HS feature extraction and classification using machine learning are summarized in Appendix: Table 6. The studies of traditional machine learning based on HS are mostly focused on feature extraction [22–24]. However, feature extraction is a critical and error-prone step. As a type of machine learning, deep learning methods can automatically learn deep features from signals and are widely used in one-dimensional physiological signals. For instance, the 1D convolutional neural networks (CNN) were proposed to learn the deep features [25] or hand-crafted features [24] of the HS and divided HS signals into normal and abnormal directly. Gated recurrent unit (GRU) is an improved recurrent neural network (RNN) proposed by Chung et al. in 2014 [26], which has a good performance in the classification and prediction of HS signals [27]. Furthermore, hybrid deep learning networks can combine the spatial features extracted by the CNN and the temporal features captured by the RNN. In [28, 29], the combination of CNN and RNN/GRU were reported to classify the HS signals and the hybrid deep learning network had better performance than the single deep learning network. Therefore, the hybrid deep learning network provides a method for predicting exercise sudden death based on HS.

In conclusion, the objectives of this study were to (1) find a suitable deep learning network to identify the HS of exercise sudden death and (2) predict the exercise sudden death in rabbits based on the process of animal experiments. The contributions of this study are as following:

1. develop a method of combining HS and deep learning to predict sudden exercise death in rabbits;
2. propose an effective deep learning network to predict sudden exercise death in rabbits.

## Results

**Fig. 1:**
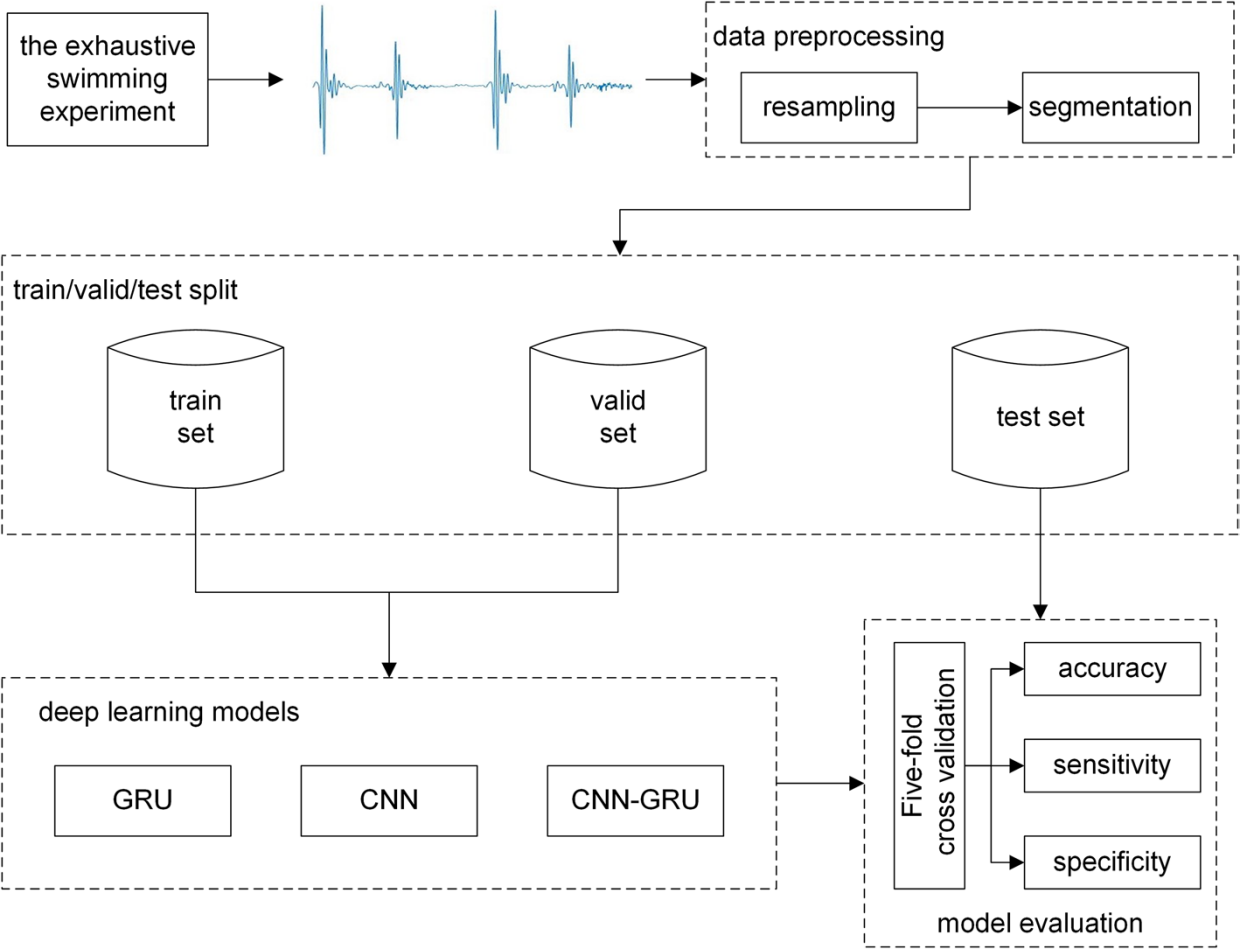
The illustration of the workflow in this paper. The CNN–GRU is the proposed network while others are the networks compared

The aim of this paper is to predict the health status (survival or exercise sudden death) of rabbits during intermittent exercise based on HS signals. The dataset consisted of the HS signals from surviving and sudden death rabbits at four different time nodes in the repeated weight-bearing exhaustive swimming experiment, and named Dataset A, Dataset B, Dataset C and Dataset D, respectively. In the classification, 80% and 20% of signals were divided into training set and test set, respectively, and then 20% of the training set were taken as validation set to monitor whether the network has been fitted. The HS signal of the same rabbit should not appear in the training set and the test set at the same time. In performance evaluation, accuracy (Acc), sensitivity (Sens), and specificity (Spec) were used to evaluation network performance. Figure 1 illustrates the framework of this paper. The algorithms of preprocessing and classification were implemented in MATLAB (version 9.5 R2018b) and python (version 3.5.4), respectively.

### The training for the CNN–GRU network

In general, the optimizer, loss function, and activation function affect network performance. The optimizer updates and calculates network parameters by minimizing the loss function, which represents the gap between prediction and actual. The activation function improves the processing of complex tasks by performing nonlinear combinations of weighted inputs. Moreover, the hyperparameters in network affect the final result, such as learning rate, dropout rate, and training epochs. The learning rate is a hyperparameter that guides how the network adjusts the weights by the gradient of the loss function, and the dropout rate is used to improve the generalization ability of the model.

In this study, the cross-entropy function with L2 norm was selected as the loss function and the regularization parameter was set to 0.001. AdamOptimizer was chosen as the optimizer due to its robustness to the choice of hyperparameters [30], ReLU was picked as the activation function of the convolution layer because it deals well with the vanishing gradient problem [31], and the learning rate and dropout rate were selected as 0.001 and 0.5, respectively.

**Fig. 2:**
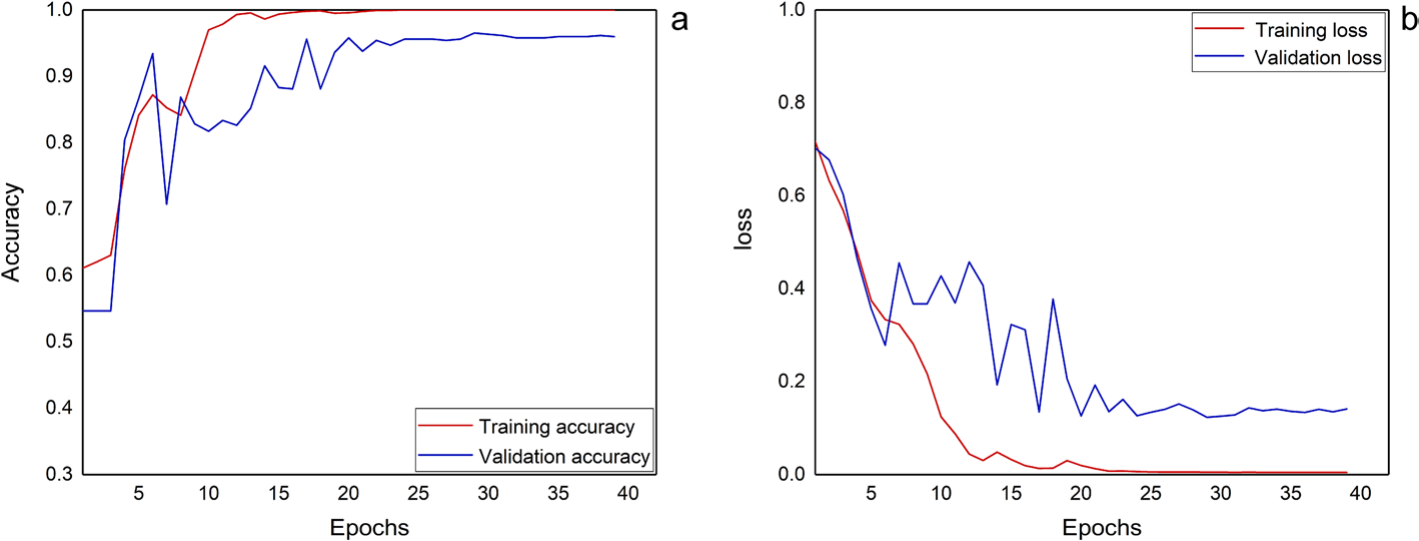
The training and validation performance of the CNN–GRU network at 50 epochs: **a** accuracy; **b** loss

The whole network was trained for 50 epochs with the batch size of 64. Here, early stopping was adopted to avoid overfitting by detecting loss values, which means first preset a number of epochs, and if the network loss value does not decrease in 10 consecutive epochs, then the network stops training. Figure 2 shows the improvement of CNN–GRU network performance with the increase of the epochs during training. The CNN–GRU network gradually converged form the 25th epoch, the accuracy and loss of the validation set gradually close to the training set, and the network stopped training at the 39th epoch, which indicates that the training epoch is preset to 50 is sufficient to make the algorithm converge.

### The performance comparison of different networks

Three different networks were used for the classification of the Dataset D. By evaluating the proposed network, CNN–GRU network got an average accuracy of 89.57%, a sensitivity of 89.38%, a specificity of 92.20%, which were 2.92%, 5.54% and 2.7% higher than CNN employed by grid search method, respectively. Moreover, the accuracy, sensitivity and specificity of the proposed network were 16.02%, 14.97% and 19.85% higher than GRU network searched by grid search method, respectively. Table 1 summarizes the performance of the three networks.

**Table 1:** The performance comparison of different networks

Networks	Acc (%)	Sens (%)	Spec (%)
CNN	86.65	83.84	89.50
GRU	73.55	74.41	72.35
Proposed network	89.57	89.38	92.20

*Acc* accuracy, *Sens* sensitivity, *Spec* specificity

### The impact of time nodes on classification results

**Fig. 3:**
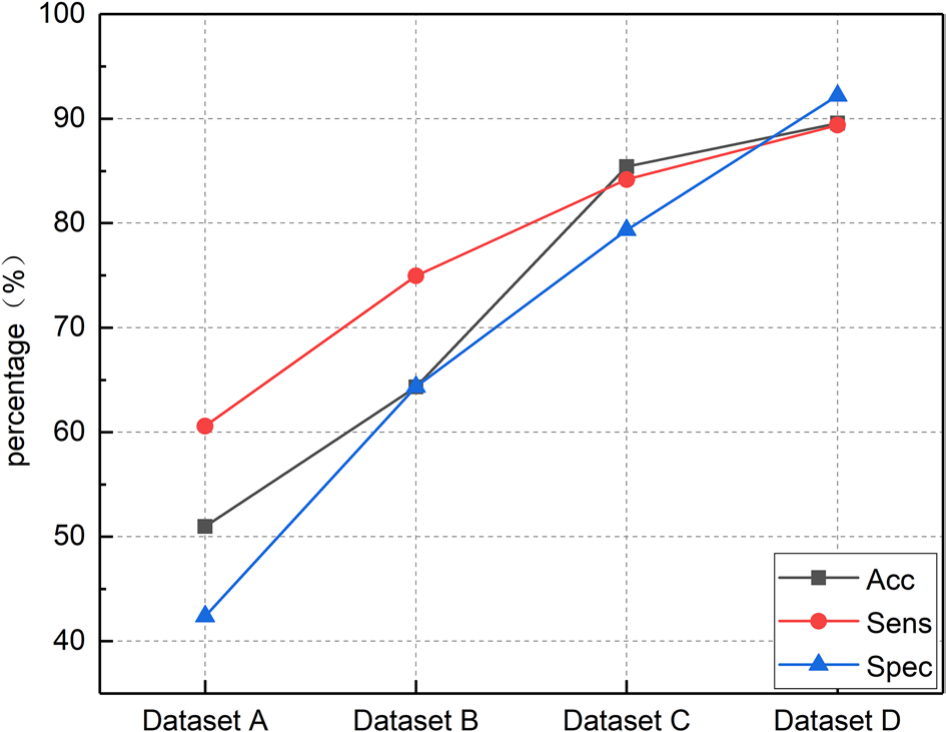
The classification results of HS signals at different time points by CNN–GRU network. When Dataset D is used as input, the CNN–GRU achieves the best performance

The HS signals at four different time nodes were fed into the CNN–GRU network to explore the law of HS changes during excessive exercise and found the time point signal that can reflect the final state. The results are shown in Table 2 and Fig. 3 describes the trend of the results. It can be found that as the experiment went on, the classification accuracy of HS gradually enhanced. Especially, when 24 h after the second exhaustion swimming (Dataset C), the network was able to recognize two classes of HS in rabbits.

**Table 2:** The performance of proposed network with four different time nodes

Dataset	Acc (%)	Sens (%)	Spec (%)
Dataset A	50.98	60.59	42.40
Dataset B	64.34	74.97	64.36
Dataset C	85.41	84.18	79.34
Dataset D	89.57	89.38	92.20

Dataset A to Dataset D, respectively, represent the HS signals at different time points in the experiment

*Acc* accuracy, *Sens* sensitivity, *Spec* specificity

## Discussion

### The comparison of different convolution kernel shapes and different numbers of layers

In order to compare the effects of different convolutional kernel shapes and different network layers on the performance of CNN–GRU, the Dataset D was used as the input of the network with convolutional kernel sizes varies in {10, 20, 30} and network layers range in {4, 6, 8} to compare its performance. Four-layer CNN–GRU network consists of a convolutional layer, a pooling layer, a GRU layer and dense layer, and the six-layer CNN–GRU network is formed by stacking a convolutional layer and a pooling layer on the four-layer CNN–GRU. The structure of the eight-layer CNN–GRU network with a convolutional kernel size of 20 is the proposed network which shown in “Methods” section. The experimental results are shown in Table 3. The results show that the best performance is obtained when the convolution kernel is chosen to be 20 and the number of network layers is chosen to be 8.

**Table 3:** Results of the different convolution kernel shapes and different numbers of convolution layers

Different layers	Convolution kernel size	Acc (%)	Sens (%)	Spec (%)
4 layers	10	72.71	80.71	74.25
	20	75.33	78.16	70.72
	30	65.77	72.15	51.38
6 layers	10	73.45	77.02	79.90
	20	77.48	79.58	77.35
	30	75.03	86.97	73.05
8 layers	10	85.35	87.91	84.75
	20	**89.57**	**89.38**	**92.20**
	30	85.72	88.29	87.42

The best result is highlighted in bold

*Acc* accuracy, *Sens* sensitivity, *Spec* specificity

### The comparison of different units

**Fig. 4:**
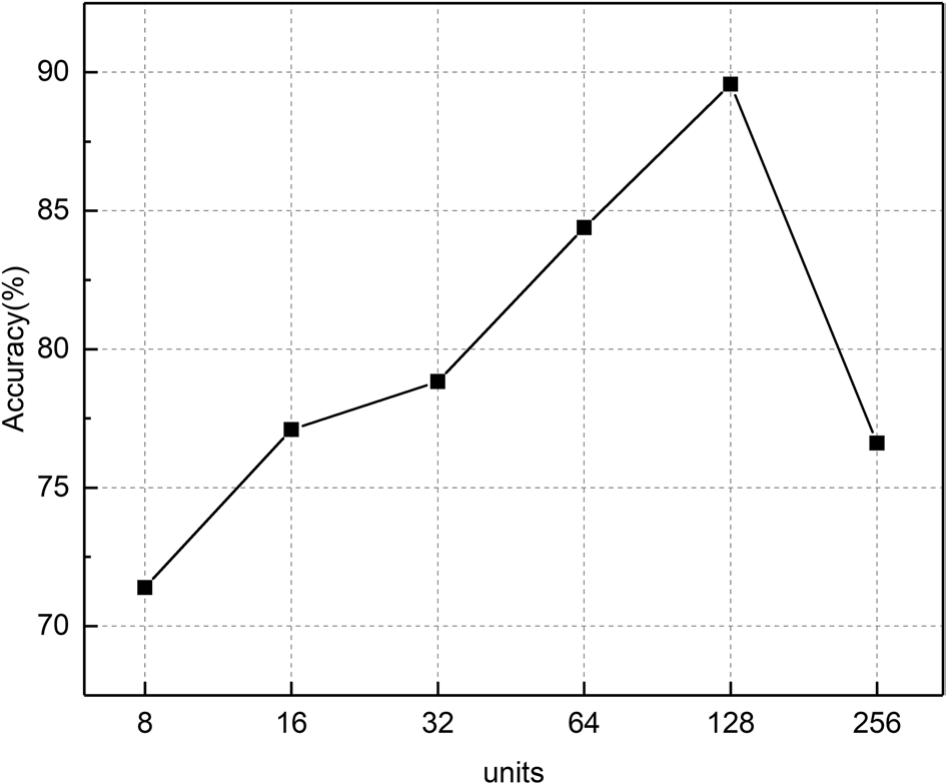
The accuracy comparison of different units in CNN–GRU. When units is set as 128, the CNN–GRU achieves the best accuracy

We also compared the impact of the number of units on the performance of CNN–GRU network. Using the structure of proposed network as a basis, the number of units varies in {8, 16, 32, 64, 128, 256} and the results are depicted in Fig. 4, it is found that the best performance is obtained when the units are 128.

### The comparison of different learning rate and dropout rate

**Fig. 5:**
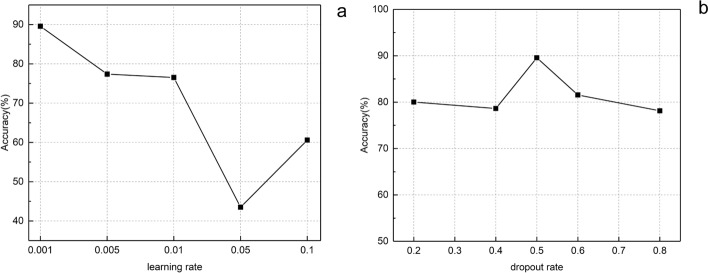
The accuracy comparison between the different learning rates and the different dropout rates: **a** learning rate; **b** dropout rate

To obtain enhanced results, based on the proposed CNN–GRU network, we tested the effects of different learning rates and dropout rates on network performance, respectively, and plotted them in Fig. 5, which shows that CNN–GRU has better performance when the learning rate is 0.001 and dropout rate is 0.5.

### The distinctions of HS characteristics between survival rabbit and exercise sudden death rabbit

**Fig. 6:**
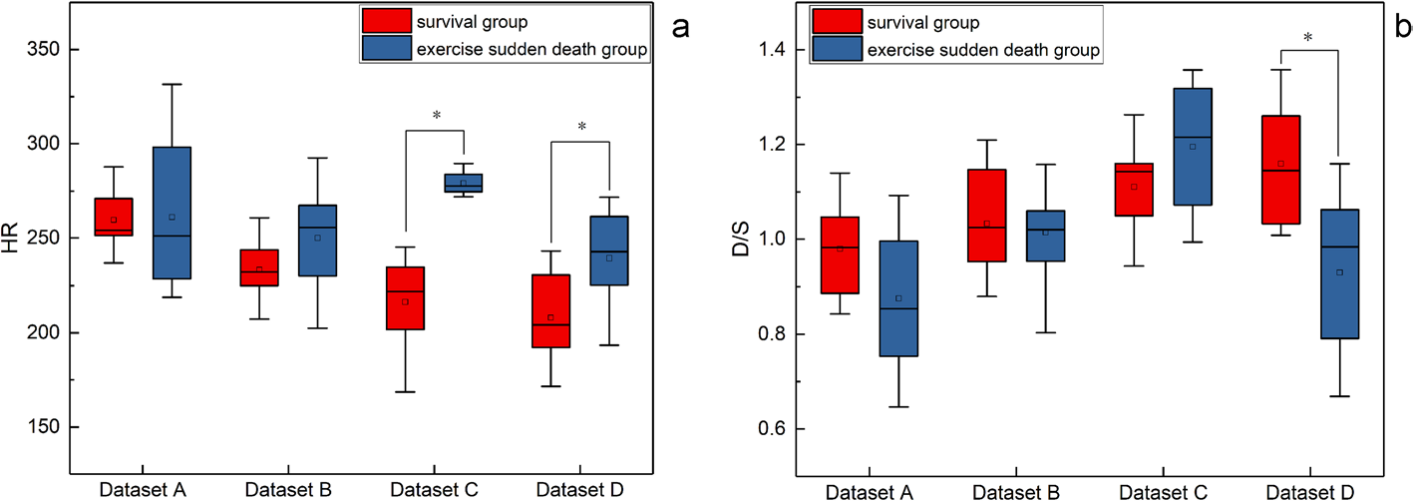
The variation of HR and D/S between survival and exercise sudden death group in different datasets: **a** shows that the HR values of Dataset C and Dataset D are different between the two groups; **b** shows that the D/S between the two groups of rabbits is different in Dataset D. *P < 0.05

Cardiac reserve indicators are often used to assess the state of cardiac function can be extracted from the HS, which is mainly composed of the first HS, the second HS. The systolic duration is the duration from the start of the first HS to the start of the second HS in a cycle, and the diastolic duration is the duration from the start of the second HS to the start of the first HS in the next cycle. The heart rate (HR) and the ratio of diastolic to systolic duration (D/S) between survival group and exercise sudden death group at different time nodes were extracted during processing, and the *t*-test was performed on SPSS (version 22.0). The results are shown in Fig. 6, and *P* values are less than 0.05 were considered significant.

Moderate exercise can improve cardiac function and reduce HR [18], and irregular HR can be a factor in screening for sudden death [32]. The exercise sudden death group had a higher HR than survival group at 24 h after the second exhausting swimming as shown in Fig. 6a. In the survival group, the HR showed a downward trend, indicating that regular physical exercise could reduce the HR, which was consistent with [18].

In addition, D/S can reflect whether cardiac muscle perfusion time during diastole is sufficient or not [33]. It has been reported that exercise-overload can proliferate collagen fibers, which limits the elongation and shortening of cardiomyocytes and increases myocardial stiffness, as a result, cardiac diastolic and systolic functions are decreased [34]. D/S in exercise sudden death group lower than in survival group at dataset D as shown in Fig. 6b, which denotes that the survival group can supply more nutrients and oxygen for systolic work because of the longer diastolic period.

### The advantages and limitations of the proposed method

The main advantages of the method proposed in this paper is that it is the first time to study exercise sudden death in rabbits using deep learning method combined with HS. The changes of cardiac function during exercise still need to be further explored, and most studies have explored the changes in cardiac function during exercise by extracting specific indicators through echocardiography, electrocardiography, blood samples, etc. In this case, the successful practice of the deep learning hybrid network proposed in this paper to predict sudden exercise death by automatically extracting depth features provides a new way to study the occurrence of sudden exercise death. However, this study has the following three limitations: (1) due to the small amount of data and the lack of database, there is no additional data available to improve the performance of the proposed method and evaluate the generalization ability of the network and pre-trained deep architecture can be considered to further handle this problem; (2) since the HS signals in the animal experiment is not continuously monitored, the HS of several rabbits that died suddenly were not collected at the moment of death, but the HS of the closest time node to the time of death were selected for follow-up work; (3) depth features lack the physical meaning that certain indicators extracted from echocardiography, electrocardiography, blood samples can express.

## Conclusion

Study of cardiac fatigue is important to prevent exercise sudden death caused by excessive exercise. Firstly, the exhaustive swimming experiment was used to collect the HS of rabbits during exercise. Secondly, the CNN–GRU network is proposed to identify survival signals and exercise sudden death signals. On this basis, two classes of HS at different time nodes were input into the network, the result shows that the 24 h after the second exhaustion swimming (Dataset C) can well reflect the final state of rabbits. Hence, we speculate that this time node may be able to predict the occurrence of exercise sudden death in rabbits. In the future work, we may combine biochemical indicators and cell analysis to further explore the pathogenesis of cardiac fatigue to exercise sudden death, and explain its changes more scientifically. In addition, we will obtain more experimental data to validate the effectiveness of the network proposed in this paper and extend the applicability of the network in cardiovascular diseases. Furthermore, we may conduct a study on human cardiac fatigue according to the findings in this paper.

## Methods

The experimental data/signals at four different time nodes were obtained through the exhaustive swimming experiment with New Zealand white rabbits, and then preprocessed it. After that, the preprocessed signals at each time node as the input of the CNN–GRU network to complete the classification and prediction of sudden exercise death.

### Animal experiments and dataset

A total of 21 New Zealand white rabbits weighing 1.7 to 2.3 kg and aged 3 to 4 months were tested in a repeated weight-bearing exhaustive swimming experiment. All procedures for experimental animals were in accordance with the National Institutes of Health guide for the care and use of Laboratory animals. This study was approved by the ethics committee of the Third Military Medical University.

**Fig. 7:**
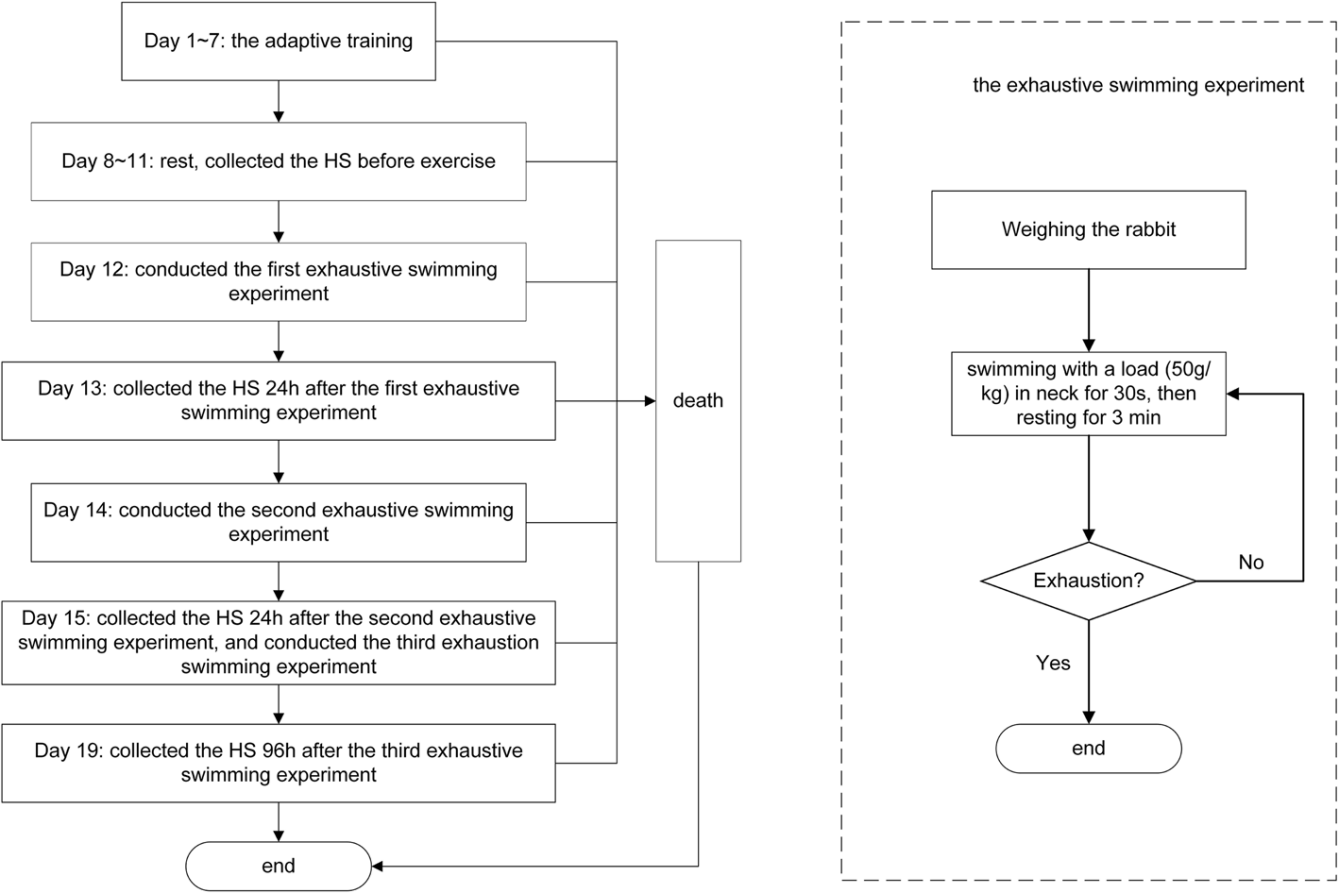
The experimental procedures of repeated weight-bearing exhaustive swimming: the left is the overall experiment process, and the right is the exhaustive swimming experiment

The specific repeated weight-bearing exhaustive swimming experimental procedures [19] were as following. Firstly, the adaptive training for 1 week was conducted on rabbits, and then, the first exhaustive swimming experiment was performed, the second exhaustive swimming experiment was conducted 48 h after the first exhaustive swimming experiment, and finally, after 24 h rest, the third exhaustive swimming experiment was carried out. Figure 7 shows the experimental process in detail. The definition of exhaustive swimming experiment is that each rabbit swims with a load (50 g/kg) for 30 s, then resting for 3 min, and then cycling to the exhaustion state, in which the rabbit's head sinks into the water for 2 s without coming to the surface. It is worth noting that the rabbits swam in an inflatable pool with the size of 700 cm × 500 cm × 70 cm, the pool was regularly watered and cleaned, and the water temperature was kept at 27 to 29 °C when swimming.

The HS signals were collected from 10 living rabbits and 11 dead rabbits. A heart sound sensor was placed at the apex of the heart for 5 min to collect heart sounds. The dataset consisted of the signals that acquired at four different time nodes of the living sample and exercise sudden death sample. According to the experimental procedures and the definition of exercise sudden death, the four different time nodes were composed of before the experiment, 24 h after the first exhaustive swimming, 24 h after the second exhaustive swimming, and 96 h after the third exhaustive swimming. If the rabbits died suddenly in the experiment, the signals included the signals at these time points before death and the signals of sudden death. In addition, since some rabbits did not collect HS at the time of sudden death, the HS closest to the time of sudden death is selected. Table 4 provides a detailed description of the HS dataset. All data used in this study were obtained from a multi-channel physiological signal acquisition system RM6240BD with XJ-102 heart sound transducer at a sampling frequency of 100 kHz and band-pass filtering frequency of 1 Hz to 10 kHz.

**Table 4:** The HS dataset collected from different time nodes

Dataset	Time node	Description
Dataset A	Pre-test signal	2482 recording form 10 survival samples and 1955 recording form 11 exercise sudden death samples
Dataset B	24 h after the first exhaustive swimming	2245 recording form 10 survival samples and 2049 recording form 10 exercise sudden death samples
Dataset C	24 h after the second exhausting swimming	2246 recording form 10 survival samples and 1317 recording form 5 exercise sudden death samples
Dataset D	96 h after the third exhausting swimming and exercise sudden death during experiment	2037 recording form 10 survival samples and 1251 recording from 11 exercise sudden death samples

### Preprocessing

#### Resampling

**Fig. 8:**
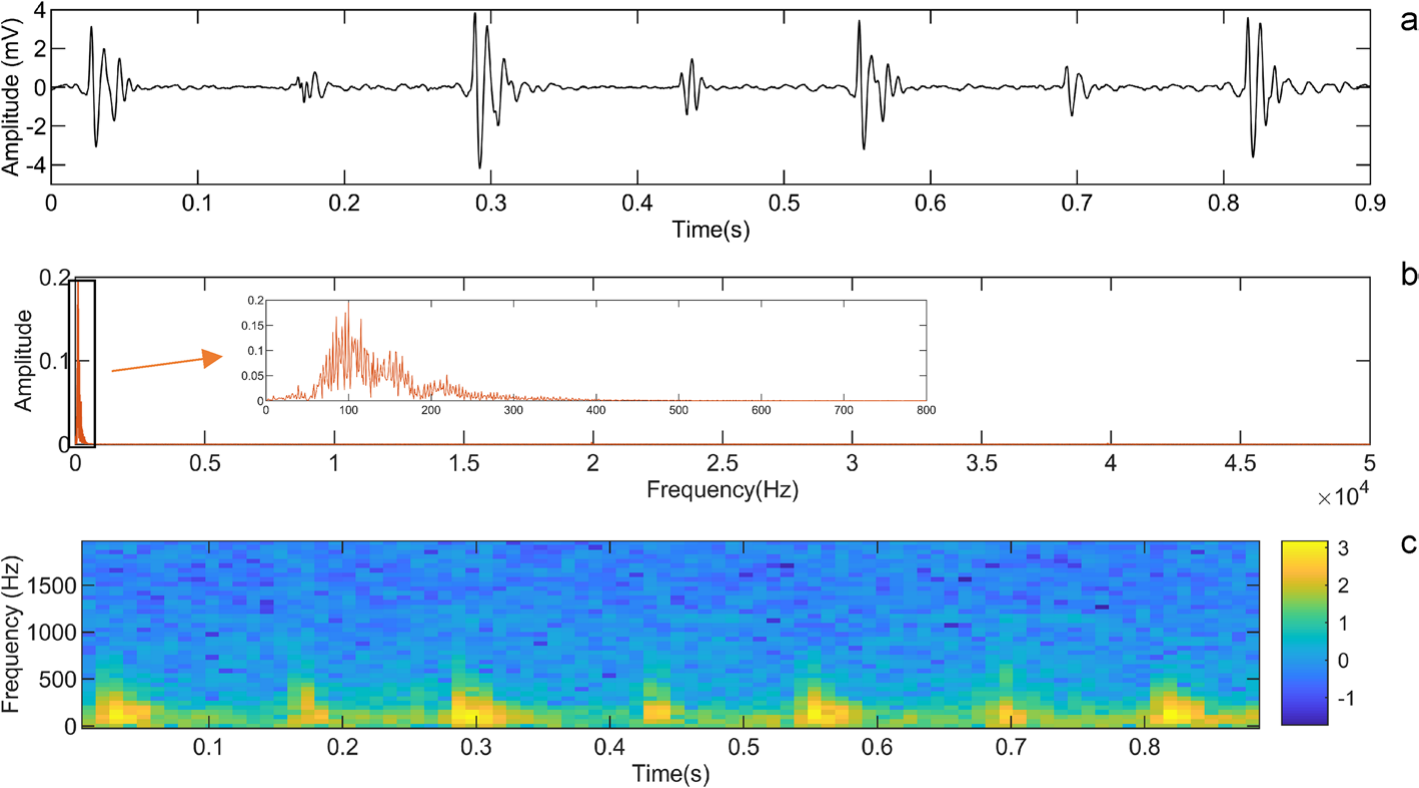
The time–frequency information of a resting New Zealand rabbit: **a** HS of a resting New Zealand Rabbit; **b** fast Fourier transform of HS; **c** short-time Fourier transform of HS

Since the high original sampling frequency may lead to an increase in computational costs, resampling according to Nyquist Sampling Theorem is an effective method. Using the theorem requires specifying the frequency range of HS in New Zealand rabbits. Fourier transform is a method for frequency domain analysis of time domain signals, which converts time domain signals into frequency domains. The fast Fourier transform reduces computation by using the butterfly operation to combine some terms of the discrete Fourier transform, a commonly used method in computers to analyze signals. Furthermore, short-time Fourier transform (STFT) [35, 36] is a commonly used method for time–frequency analysis, which characterizes the signal at a certain time by a time window. In this study, we used the fast Fourier transform and STFT to analyze the time–frequency domain of rabbit HS, and found that the frequencies of HS and major components in New Zealand rabbits were within 1000 Hz. In STFT, the Hanning window was used as the window function, the window width was 2048, and the overlap points were 1024. Figure 8 shows the time–frequency information of heart sounds in a resting New Zealand rabbit. Therefore, the signals were resampled to 2000 Hz according to the Nyquist Sampling Theorem.

#### Segmentation

In order to ensure that each signal had the same length, we employed Viola integral method [37] and the normalized Shannon energy method [38] to extract envelope, and then selected the double threshold [39] method to locate and segment the HS signal. In contrast to the currently widely used logistic regression-based hidden semi-Markov model [40], this method can mark the HS time domain features without reference to electrocardiogram and the steps were as follows:

1. set the time scale:
  1 $$ L_{{\text{T}}} = 0.5 \times s \times {\text{Fs,}} $$
  where $s$ is the minimum duration of S1 and ${\text{Fs}}$ is the sampling frequency, which were set as 0.02 and 2000, respectively.
2. obtain the signal mean sequence:
  2 $$ \overline{X}_{{\text{T}}} \left( m \right) = \frac{1}{{2L_{{\text{T}}} + 1}}\sum\limits_{{k = m - L_{{\text{T}}} }}^{{m + L_{{\text{T}}} }} {X_{{\text{T}}} \left( k \right)} , $$
  where $m = L_{{\text{T}}} ,L_{{\text{T}}} + 1, \ldots ,M - 1 - L_{{\text{T}}}$, *M* is the length of original HS signal.
3. calculate the characteristic envelope of the Viola integral waveform:
  3 $$ E_{{\text{T}}} \left( m \right) = \frac{1}{{2L_{{\text{T}}} + 1}}\sum\limits_{{k = m - L_{{\text{T}}} }}^{{m + L_{{\text{T}}} }} {\left[ {X_{{\text{T}}} \left( k \right) - \overline{X}_{{\text{T}}} \left( m \right)} \right]^{2} } . $$
4. calculate the mean Shannon entropy and normalize it according [38].
5. locate the S1 onsets by the double threshold method, in which the larger threshold was $H = M \times a$, and the smaller one was $L = M \times b$, where *M* is the mean value of envelope, the value of $a$ varies from 0.6 to 1.1, and the value of $b$ varies from 0.01 to 0.03, which can be adjusted according to the specific situation of the signal.

**Fig. 9:**
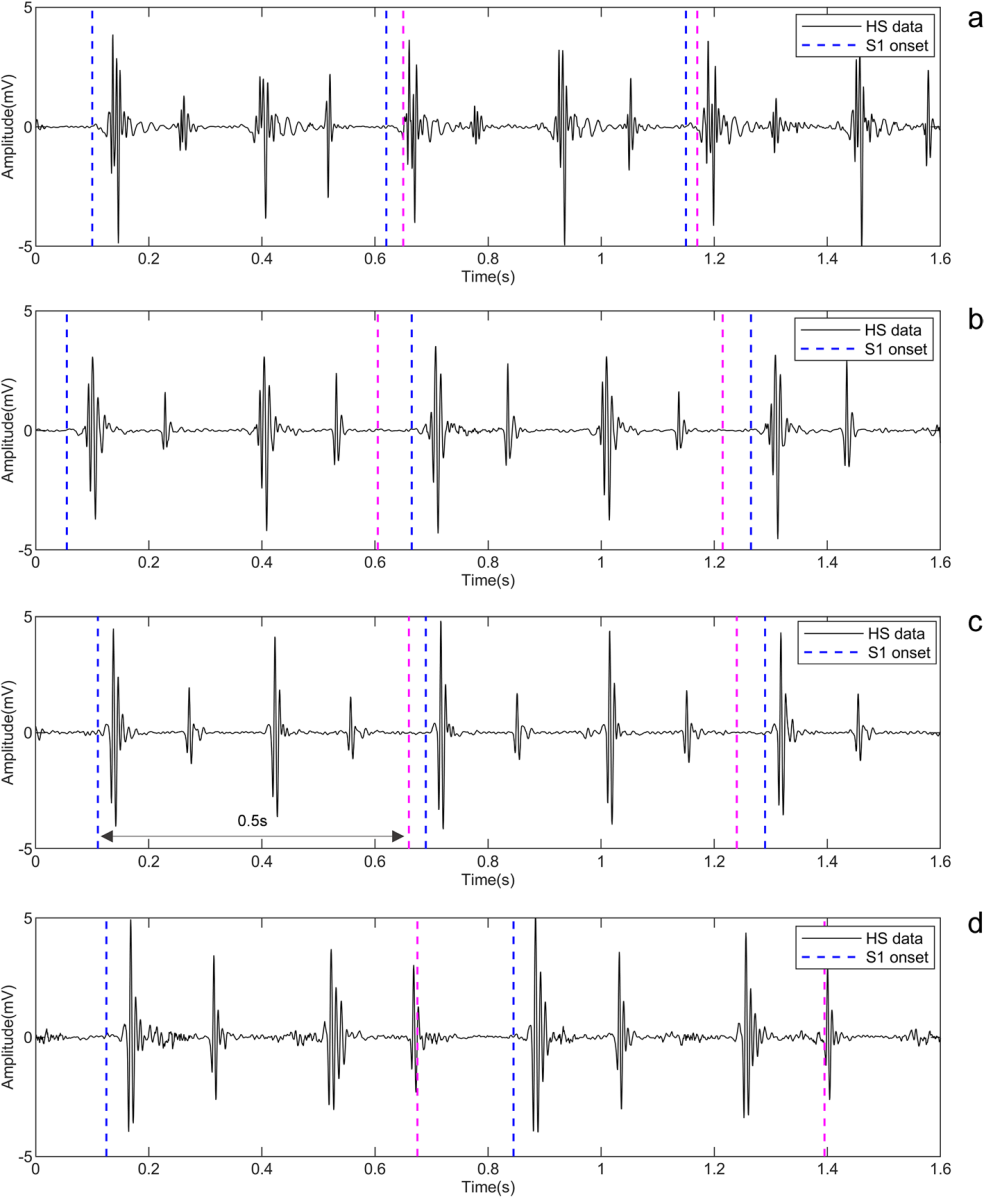
The location and segmentation of HS in a rabbit at four different time points: **a** before the experiment; **b** 24 h after the first exhaustive swimming; **c** 24 h after the second exhaustive swimming; **d** 96 h after the third exhaustive swimming. The blue and magenta dashed lines indicate the start and end of segmentation, respectively

In this work, the S1 onsets were taken as the starting point of segmentation, and then a 0.5 s signal was selected for segmentation, the S1 onset marking and segmentation strategies can be seen in Fig. 9. Finally, 4437, 4294, 3563 and 3288 recordings from four time points in the rabbit experiment were obtained, which, respectively, constituted dataset A, B, C and D, and are summarized in Table 4.

### The proposed network

**Fig. 10:**
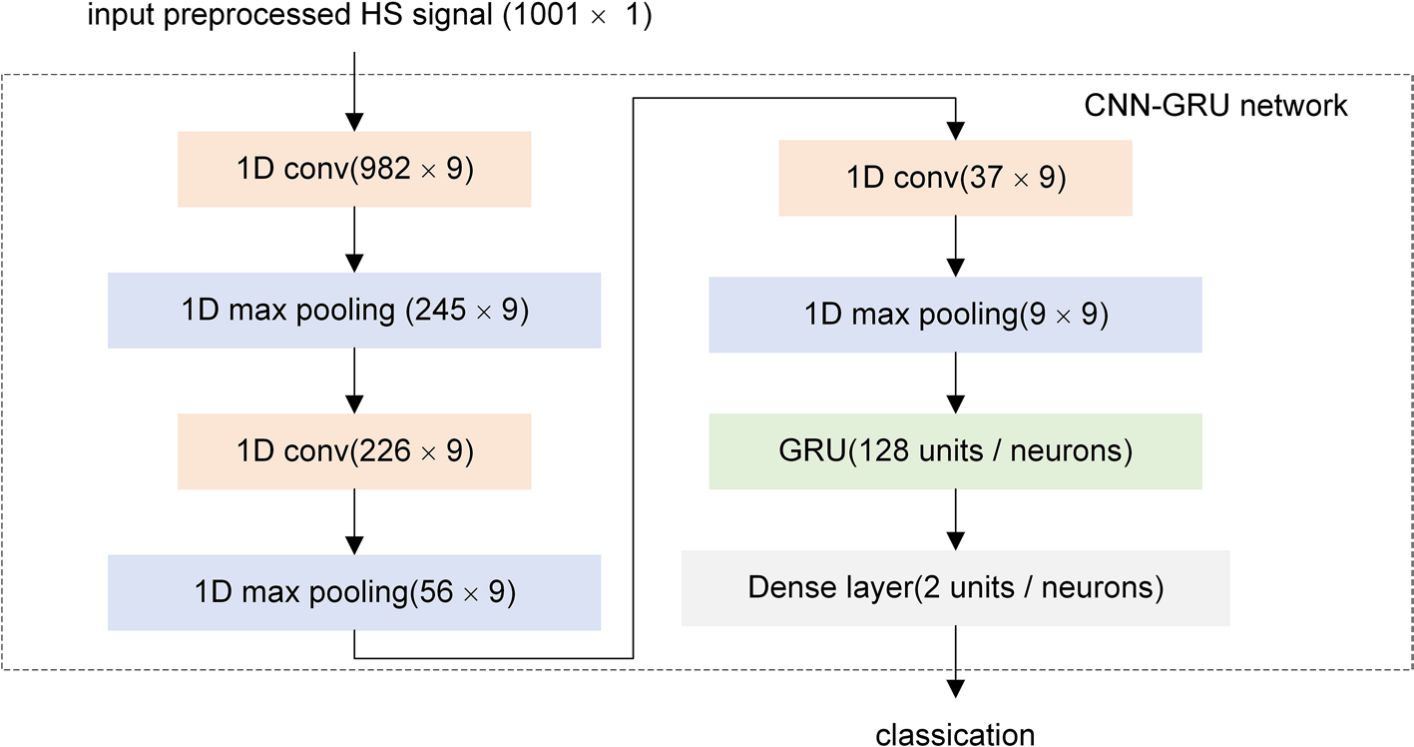
The structure of the proposed network

Generally, the hybrid network of deep learning has better performance in classification and prediction because they can combine the strengths of different deep learning networks [28, 41, 42]. Therefore, a CNN–GRU deep learning network was proposed by grid search method in this study. Figure 10 describes the structure of the network and the detailed information is shown in Table 5. The first six layers of the CNN–GRU network were the cross-connected convolutional layers and the max pooling layers, and the seventh layer was a single GRU layer with 128 units, followed by the dense layer for classification. When HS signals of 1001 × 1 were fed into CNN–GRU, the detailed classification process is as follows:

- layer 1: a convolutional layer with kernels size of 20, number of filters is 9 and stride of 1 with valid padding. Output shape is (982, 9);
- layer 2: a max pooling layer with pool size of 4 and stride of 4. Output shape is (245, 9);
- layer 3: a convolutional layer with kernels size of 20, number of filters is 9 and stride of 1 with valid padding. Output shape is (226, 9);
- layer 4: a max pooling layer with pool size of 4 and stride of 4. Output shape is (56, 9);
- layer 5: a convolutional layer with kernels size of 20, number of filters is 9 and stride of 1 with valid padding. Output shape is (37, 9);
- layer 6: a max pooling layer with pool size of 4 and stride of 4. Output shape is (9, 9);
- layer 7: a GRU layer of 128 units with dropout of 0.5. Output shape is (128);
- layer 8: a dense layer of 2 output units with softmax activation function. Output shape is (2). 2 classification classes of HS signals in rabbits of survival or exercise sudden death.

**Table 5:** The detailed information of the proposed network

Layers	Layers types	Output size	Kernel/pool size	Filter numbers	Stride	Activation function
0	Input	1001 × 1	–	–	–	–
1	1D conv	982 × 9	20	9	1	ReLU
2	1D max pooling	245 × 9	4	–	4	–
3	1D conv	226 × 9	20	9	1	ReLU
4	1D max pooling	56 × 9	4	–	4	–
5	1D conv	37 × 9	20	9	1	ReLU
6	1D max pooling	9 × 9	4	–	4	–
7	GRU	128	–	–	–	dropout = 0.5
8	dense	2	–	–	–	softmax

### Performance

In this paper, fivefold cross-validation was used to evaluate network and the performance of each fold was evaluated based on Acc, Sens and Spec. These indices can be calculated as follows:

4 $$ {\text{Acc}} = \frac{{{\text{TP}} + {\text{TN}}}}{{{\text{TP}} + {\text{TN}} + {\text{FP}} + {\text{FN}}}}, $$

5 $$ {\text{Sens}} = \frac{{{\text{TP}}}}{{{\text{TP}} + {\text{FN}}}}, $$

6 $$ {\text{Spec}} = \frac{{{\text{TN}}}}{{{\text{TN}} + {\text{FP}}}}, $$

where ${\text{TP}}$ is the true positive, ${\text{TN}}$ is the true negative, ${\text{FP}}$ is the false positive, and ${\text{FN}}$ is the false negative.

## Data Availability

Due to the benefit of the National Natural Science Foundation of China, the HS database used in this study cannot be published, and the code can be requested from the corresponding author.

## Appendix

See Table 6.

**Table 6:** Studies for HS feature extraction and classification using machine learning

Year	Author	Dataset	Feature extraction methods	Classifier	Results		
					Sens (%)	Spec (%)	Acc (%)
2015	Zheng et al. [23]	88 normal heart sounds, 64 abnormal heart sounds	MF-DFA, MESE, EMD	HMM	82.95	79.68	81.58
				BP-ANN	85.23	82.81	84.21
				LS-SVM	96.59	93.75	95.39
2016	Thomae et al. [29]	PhysioNet	1D CNN	Bidirectional GRU	96	83	–
2016	Potes et al. [43]	PhysioNet	LR-HMMS, MFCC	AdaBoost	70	88	–
			Frequency bands decomposition	CNN	79	86	–
2019	Li et al. [25]	2532 recordings from healthy subjects, 664 recordings from patients	DAE	1D CNN	–	–	97.85
			MFCC	1D CNN	–	–	91.02
2020	Li et al. [24]	PhysioNet	Eight domains	CNN	87	86.6	86.8
2020	Gao et al. [27]	1286 normal recordings form PhysioNet, 108 abnormal heart sounds from patients	–	SVM	–	–	87.62
				FCN	–	–	94.65
				LSTM	–	–	96.29
				GRU	–	–	98.82
2020	Deng et al. [28]	PhysioNet	MFCC	CRNN	98.66	98.01	98.34
				PRCNN	97.33	97.33	97.34

*MF-DFA* multifractal detrended fluctuation analysis, *MESE* maximum entropy spectra estimation, *EMD* empirical mode decomposition, *CNN* convolutional neural network, *1D CNN* one-dimensional convolutional neural network, *DAE* denoising autoencoder, *MFCC* Mel-frequency cepstrum coefficient, *HMM* hidden Markov model, *LR-HSMM* logistic regression-based hidden semi-Markov model, *BP-ANN* back-propagation artificial neural network, *LS-SVM* least square support vector machine, *GRU* gated recurrent unit, *FCN* Fully Convolutional Network, *LSTM* long-short term memory network, *CRNN* convolutional recurrent neural networks, *PRCNN* paralleling recurrent convolutional neural network
